# Biocompatibility of Mineral Trioxide Aggregate with TiO2 Nanoparticles on Human Gingival Fibroblasts

**DOI:** 10.4317/jced.53126

**Published:** 2017-02-01

**Authors:** Mohammad Samiei, Negin Ghasemi, Marzieh Aghazadeh, Baharak Divband, Farzaneh Akbarzadeh

**Affiliations:** 1 Associate Professor, Department of Endodontics, Dental Faculty, Tabriz University (Medical Sciences), Tabriz, Iran; 2 Assistant Professor, Department of Endodontics, Dental and Periodontal Research Center, Dental Faculty, Tabriz University (Medical Sciences), Tabriz, Iran; 3 Assistant Professor, Department of Oral Medicine , Dental Faculty, Tabriz University (Medical Sciences), Tabriz, Iran; 4 Assistant Professor, Department of Chemistry, Tabriz University, Tabriz, Iran; 5 Privaite Practice, Tabriz, Iran

## Abstract

**Background:**

The New compositions of white mineral trioxide aggregate (WMTA) or use of various additives like nanoparticles might affect MTA’s ideal characteristics This study was performed to evaluate the cytotoxicity of WMTA and WMTA with Titanium dioxide (TiO2) nanoparticles (1% weight ratio) at different storage times after mixing on human gingival fibroblasts (HGFs).

**Material and Methods:**

HGFs were obtained from the attached gingiva of human premolars. HGFs were cultured in Dulbecco’s Modified Eagle medium, supplemented with 10% fetal calf serum, penicillin and streptomycin. The cells were exposed to WMTA (groups 1 and 2) and WMTA+TiO2 (groups 3 and 4). The fifth and sixth groups served as controls. Each group contained 15 wells. After 24h (groups 1, 3 and 5) and 48 h (groups 2, 4 and 6) of exposure, HGF viability was determined by Mosmann’s tetrazolium toxicity (MTT) assay. Statistical analysis of the data was performed by using one-way analysis of variance and Tukey post hoc test, with significance of *p* < 0.05.

**Results:**

With both materials, the viability of HGFs significantly decrased with increasing the incubation time from 24h to 48 h (*P*<0.05). There was no significant difference between the materials regarding HGF viability (*P*>0.05).

**Conclusions:**

Under the limitations of the present study, incorporation of TiO2 nanoparticles into MTA at 1 wt% had no negative effect on its biocompatibility.

** Key words:**Cytotoxicity, fibroblast, MTA, MTT assay, nanoparticle, TiO2.

## Introduction

Mineral trioxide aggregate (MTA) is the biomaterial of choice for use in many challenging endodontic procedures such as apexogenesis, apexification, perforation repair and apical surgery hoping that it will promote healing of pulpal and periradicular tissues (1,2). Although MTA as a biomaterial is widely used since its introduction into the endodontic practice, it is not an easy material to handle. The long setting time of MTA make it inappropriate for one-appointment treatment modalities (3,4).

Some of the attempts to improve the properties of MTA include incorporation of silver and zinc nanoparticles into it. Titanium dioxide nanoparticles, too, have drawn attention in the dental materials field. TiO2 nanoparticles have wide industrial applications as in pharmaceuticals, pigments and cosmetics. It is also widely used in the biomedical field for integration into the osseous tissue (5-8).

Its incorporation into glass-ionomer and acrylic resin has resulted in the improvement of the physical properties of these materials in a concentration-dependent manner. In addition, mouthrinses containing these nanoparticles exhibit antibacterial activity against *S. mutans* and *S. Sanguis* (6-11). In a study, incorporation of TiO2 nanoparticles to white Portland cement resulted in an increase in its flexural and compressive strengths and in a decrease in its setting time (12). Given the similarities between the chemical structures of MTA and Portland cement (2), it is expected that incorporation of these nanoparticles into MTA will result in improvements in its properties, too.

However, new compositions of MTA or use of various additives might affect MTA’s ideal characteristics, which should be supported by comprehensive investigations. During vital pulp therapy, perforation repair and retrograde filling, the cytotoxicity of the material used might influence the viability of periradicular cells, resulting in cell death by apoptosis or necrosis (1,13). Therefore, it is important to avoid dental materials that are toxic to the pulpal and periapical cells.

One of the cells that have drawn attention in cytotoxicity studies is the fibroblast which is the main cell involved in the synthesis and formation of collagen fibers and mesenchymal cells that can be differentiated into cementoblasts and osteoblasts (1).

The cytotoxicity of MTA has been investigated in many studies which have demonstrated its biocompatibility and good biologic properties (14-18). The toxicity of titanium dioxide nanoparticles on monoblastoids, lymphocytes, erythrocytes and keratinocytes has been evaluated and the results have shown dose-dependent necrosis and apoptosis in the cells (5,7,19-21). However, no studies are available on the biocompatibility of these nanoparticles in combination with endodontic biomaterials with dental tissue cells.

The aim of this study was to evaluate biocompatibility of MTA mixed with TiO2 nanoparticles at 1 wt% with human gingival fibroblasts at 48- and 72-hour exposure times.

## Material and Methods

The design of this study was approved in investigation committee of deputy dean for research (No 56-2716).

-Cell culture 

After obtaining informed consents, human gingival fibroblasts (HGFs) were harvested from inflammation-free attached gingiva (inclusion criteria) of premolar teeth from ten healthy individuals who underwent orthodontic extractions. The epithelium of the gingiva was detached from the gingival connective tissue and immediately transferred into Hank’s balanced salt solution (HBSS; Sigma-Aldrich, St Louis, MO, USA), containing penicillin (100 U/mL), streptomycin (100 g/mL) and amphotericin B (5 g/mL). The explants were cut into very small pieces, rinsed in Dulbecco’s modified Eagle medium (DMEM; Gibco Laboratories, Grand Is., NY, USA) twice and left to attach to the bottom of 25-cm2 tissue culture flasks (Corning, NY, USA). DMEM, supplemented with 10% fetal calf serum, penicillin (100 U/mL) and streptomycin (5 g/mL), was transferred into the flasks and incubated at 37°C under 95% air/5% CO2 condition. Fibroblasts isolated from the fourth and fifth passages were evaluated in this study.

-Sample preparation

The materials tested in this study consisted of WMTA (Angelus Dental Industry Products, Londrina, Brazil) and WMTA with 1% weight ratio of TiO2 nanoparticles. The materials were mixed under aseptic conditions with a powder-to-liquid ratio of 3:1. Thirty disks (15 in each group), measuring 5 mm in diameter and 3 mm in thickness, were prepared from each material. The disks were wrapped in moist pieces of gauze and incubated for 24 h in a closed container. Then in the relevant plate of each group, WMTA in groups 1 and 2 and WMTA+TiO2 in groups 3 and 5, a disk (n=15 for each well) of biomaterial was placed in each well in close proximity to fibroblasts for 24 and 48 h, during which the cell culture plates were incubated at 37°C.

-Cytotoxicity assay

In order to evaluate the cytotoxic effects of the materials used in this study, 3-{4,5-dimethylthiazol-2-yl}-2,5-diphenyl tetrazolium bromide colorimetric assay [Mosmann’s tetrazolium toxicity (MTT) assay] was used and reported as the percentage of viable cells for each well. After 4 hours of incubation, the plates were centrifuged and the culture medium was removed. Precipitated formazan crystals were dissolved by adding 200 μL of solvent (dimethyl sulfoxide) to each well. The microplates were shaken at room temperature for 10 min and prepared for reading by a microplate reader at 570 nm. The percentage of metabolic activity was calculated using the formula: (Test optical density/controloptical density)*100.

-Statistical analysis

Statistical analysis of the data was performed by using one-way analysis of variance and Tukey post hoc test, with significance of *p* < 0.05.

## Results

**Figure 1:**
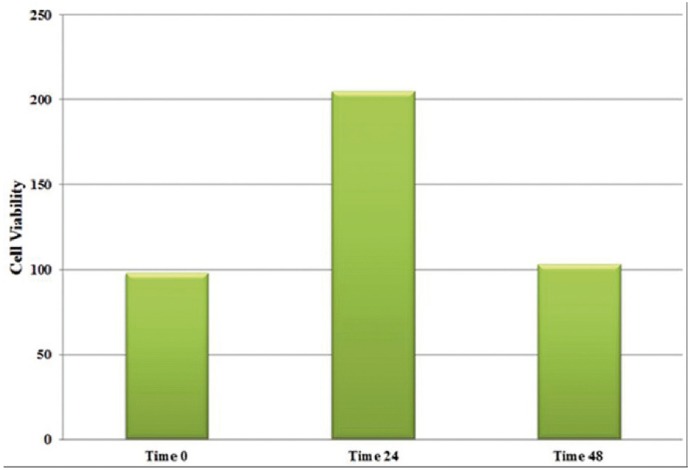
The cell viability of HGFs in presence of WMTA.

**Figure 2:**
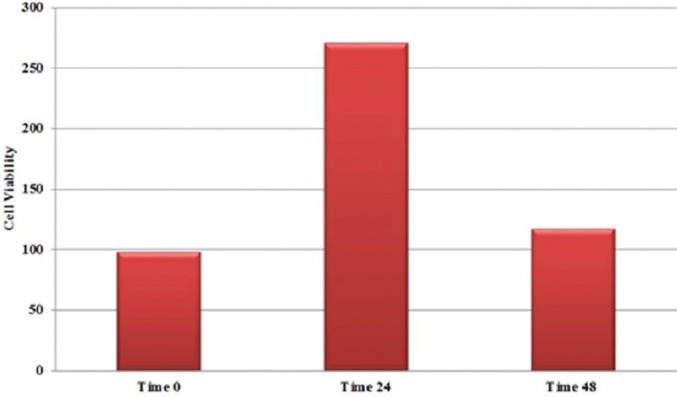
The cell viability of HGFs in presence of WMTA+TiO2.

Cell viability of HGFs after 24 and 48 h of incubation in both of the study materials are shown in figures 1 and 2. Maximum and minimum cell viability was shown in MTA+TiO2/24h and MTA/48h; respectively. The effect of the incubation period on the cell viability was significant (*p*=0.02); but not for the type of the material (MTA vs. MTA+TiO2) (*p*=0.09). There was no significant difference in HGFs between the test groups at both incubation intervals (*p*=0.08 for 24 h, *p*=0.07 for 48 h). In both test groups, the viability of HGFs decreased significantly with increasing the incubation time (*p*=0.03 for WMTA, *p*=0.01 for WMTA/TiO2).

## Discussion

The aim of this study was to evaluate the effect of incorporation of TiO2 on the biocompatibility of MTA; the results showed that the presence or absence of these nanoparticles had no effect on the viability of fibroblasts.

The most important requirement for better efficacy of biomaterials like MTA is their biocompatibility with the host tissue upon implantation (13,14). The clinical success of a biomaterial depends upon the ability to produce the required effect of cellular or tissue response (15,22).

To date, a wide array of methods, both *in vivo* and *in vitro*, have been used to evaluate the biocompatibility of filling materials. Primary cells derived from living tissues are required for specific sensitivity testing; human gingival fibroblasts were selected in the present study to simulate the clinical conditions (1).

The periodontal ligament is a connective tissue around the tooth root and bone. This tissue is continuous with the gingival connective tissue and consists of fibroblasts that are the principal cells producing and forming collagen fibers and mesenchymal cells with the capacity to differentiate into cementoblasts and osteoblasts. Therefore, the primary gingival fibroblasts are appropriate for the evaluation of the toxicity of this material (13,23,24). On the other hand, fibroblasts are the most abundant cells of the pulp and periodontal ligament connective tissue (1); therefore, during procedures such as vital pulp therapy, apexification and during their use as a retrofilling materials during apicoectomy procedures, MTA comes into contact with these cells. In this study, *in vitro* cytotoxic effects of tested materials on gingival fibroblasts were analyzed using MTT assay because of its simple, precise and accessible nature (1). MTT is a colorimetric assay based on the ability of mitochondrial dehydrogenase enzymes in living cells to convert the yellow water-soluble tetrazolium salt into dark blue formazan crystals. The amount of formazan produced is directly proportional to the number of viable cells. Its advantage is that the formazan crystals are soluble in tissue culture media and therefore there is no need for the dissolving procedure (25-27).

Metal nanoparticles have long been used in medicine because of their bactericidal and bacteriostatic effects (28). Nanotechnology has found applications in the field of dental materials in recent years and nanoparticles have been introduced into the structure of dental materials. However, many of the properties of metal nanoparticles are still to be elucidated. For example, cytotoxic properties of nanoparticles still need further research (9,22).

In the present study, TiO2 nanoparticles were used in combination with MTA at 1 wt%. Based on the results of a study by Khataee *et al.*, incorporation of this percentage of nanoparticles into white Portland cement improved some of its physical properties (12) and it is believed that such an effect can be expected with MTA, too. However, further studies are necessary to substantiate such a hypothesis. Since no similar studies are available in relation to the cytotoxicity of MTA in combination with TiO2 nanoparticles, it is not possible to compare the results of this study with other studies. Under the limitations of this study, incorporation of titanium dioxide nanoparticles into MTA had no negative effect on its biocompatibility.

## References

[1] Ghasemi N, Rahimi S, Lotfi M, Solaimanirad J, Shahi S, Shafaie H (2014). Effect of Mineral Trioxide Aggregate, Calcium-Enriched Mixture Cement and Mineral Trioxide Aggregate with Disodium Hydrogen Phosphate on BMP-2 Production. Iran Endod J.

[2] Parirokh M, Torabinejad M (2010). Mineral trioxide aggregate: a comprehensive literature review--Part I: chemical, physical, and antibacterial properties. J Endod.

[3] Shahi S, Ghasemi N, Rahimi S, Yavari H, Janani M, Mokhtari H (2015). The Effect of Different Mixing Methods on Working Time, Setting Time, Dimensional Changes and Film Thickness of Mineral Trioxide Aggregate and Calcium-Enriched Mixture. Iran Endod J.

[4] Lotfi M, Ghasemi N, Rahimi S, Bahari M, Vosoughhosseini S, Saghiri MA (2014). Effect of smear layer on the push-out bond strength of two endodontic biomaterials to radicular dentin. Iran Endod J.

[5] Bayat N, Lopes VR, Scholermann J, Jensen LD, Cristobal S (2015). Vascular toxicity of ultra-small TiO2 nanoparticles and single walled carbon nanotubes in vitro and in vivo. Biomaterials.

[6] Garcia-Contreras R, Scougall-Vilchis RJ, Contreras-Bulnes R, Sakagami H, Morales-Luckie RA, Nakajima H (2015). Mechanical, antibacterial and bond strength properties of nano-titanium-enriched glass ionomer cement. J Appl Oral Sci.

[7] Guglielmotti MB, Domingo MG, Steimetz T, Ramos E, Paparella ML, Olmedo DG (2015). Migration of titanium dioxide microparticles and nanoparticles through the body and deposition in the gingiva: an experimental study in rats. Eur J Oral Sci.

[8] Shirkavand S, Moslehifard E (2014). Effect of TiO2 Nanoparticles on Tensile Strength of Dental Acrylic Resins. J Dent Res Dent Clin Dent Prospects.

[9] Samiei M, Farjami A, Dizaj SM, Lotfipour F (2016). Nanoparticles for antimicrobial purposes in Endodontics: A systematic review of in vitro studies. Mater Sci Eng C Mater Biol Appl.

[10] Tomankova K, Horakova J, Harvanova M, Malina L, Soukupova J, Hradilova S (2015). Cytotoxicity, cell uptake and microscopic analysis of titanium dioxide and silver nanoparticles in vitro. Food Chem Toxicol.

[11] Ahrari F, Eslami N, Rajabi O, Ghazvini K, Barati S (2015). The antimicrobial sensitivity of Streptococcus mutans and Streptococcus sangius to colloidal solutions of different nanoparticles applied as mouthwashes. Dent Res J.

[12] Khataee R, Heydari V, Moradkhannejhad L, Safarpour M, Joo SW (2013). Self-cleaning and mechanical properties of modified white cement with nanostructured TiO2. J Nanosci Nanotechnol.

[13] Garcia Lda F, Santos AD, Moraes JC, Costa CA (2016). Cytotoxic effects of new MTA-based cement formulations on fibroblast-like MDPL-20 cells. Braz Oral Res.

[14] Chang SW, Kim JY, Kim MJ, Kim GH, Yi JK, Lee DW (2016). Combined effects of mineral trioxide aggregate and human placental extract on rat pulp tissue and growth, differentiation and angiogenesis in human dental pulp cells. Acta Odontol Scand.

[15] Küçükkaya S, Görduysus MÖ, Zeybek ND, Müftüoðlu SF (2016). In Vitro Cytotoxicity of Calcium Silicate-Based Endodontic Cement as Root-End Filling Materials. Scientifica.

[16] Oh S, Perinpanayagam H, Lee Y, Kum JW, Yoo YJ, Lim SM (2016). Effect of acidic solutions on the microhardness of dentin and set OrthoMTA and their cytotoxicity on murine macrophage. Restor Dent Endod.

[17] Rodriguez-Lozano FJ, Garcia-Bernal D, Onate-Sanchez RE, Ortolani-Seltenerich PS, Forner L, Moraleda JM (2015). Evaluation of cytocompatibility of calcium silicate-based endodontic sealers and their effects on the biological responses of mesenchymal dental stem cells. Int Endod J.

[18] Saberi EA, Karkehabadi H, Mollashahi NF (2016). Cytotoxicity of Various Endodontic Materials on Stem Cells of Human Apical Papilla. Iran Endod J.

[19] Besinis A, De Peralta T, Tredwin CJ, Handy RD (2015). Review of nanomaterials in dentistry: interactions with the oral microenvironment, clinical applications, hazards, and benefits. ACS nano.

[20] Garcia-Contreras R, Sugimoto M, Umemura N, Kaneko M, Hatakeyama Y, Soga T (2015). Alteration of metabolomic profiles by titanium dioxide nanoparticles in human gingivitis model. Biomaterials.

[21] Rajapakse K, Drobne D, Kastelec D, Kogej K, Makovec D, Gallampois C (2016). Proteomic analyses of early response of unicellular eukaryotic microorganism Tetrahymena thermophila exposed to TiO particles. Nanotoxicology.

[22] Samiei M, Aghazadeh M, Lotfi M, Shakoei S, Aghazadeh Z, Vahid Pakdel SM (2013). Antimicrobial Efficacy of Mineral Trioxide Aggregate with and without Silver Nanoparticles. Iran Endod J.

[23] Rathinam E, Rajasekharan S, Chitturi RT, Martens L, De Coster P (2015). Gene Expression Profiling and Molecular Signaling of Dental Pulp Cells in Response to Tricalcium Silicate Cements: A Systematic Review. J Endod.

[24] Slompo C, Peres-Buzalaf C, Gasque KC, Damante CA, Ordinola-Zapata R, Duarte MA (2015). Experimental Calcium Silicate-Based Cement with and without Zirconium Oxide Modulates Fibroblasts Viability. Braz Dent J.

[25] Gomes-Filho JE, Sivieri-Araujo G, Sipert CR, da Silva Santos LM, de Azevedo Queiroz IO, Men Martins C (2016). Evaluation of photodynamic therapy on fibroblast viability and cytokine production. Photodiagnosis Photodyn Ther.

[26] Li R, Zhang Q (2016). The expression of serine protease HtrA1 in human periodontal ligament tissue and the effect of HtrA1 on the proliferation of human periodontal ligament cells. Zhonghua Kou Qiang Yi Xue Za Zhi.

[27] Silva EJ, Senna PM, De-Deus G, Zaia AA (2016). Cytocompatibility of Biodentine using a three-dimensional cell culture model. Int Endod J.

[28] Samiei M, Ghasemi N, Divband B, Balaei E, Hosien Soroush Barhaghi M, Divband A (2015). Antibacterial efficacy of polymer containing nanoparticles in comparison with sodium hypochlorite in infected root canals. Minerva stomatol.

